# Waste Autochthonous Tuscan Olive Leaves (*Olea europaea var. Olivastra seggianese*) as Antioxidant Source for Biomedicine

**DOI:** 10.3390/ijms20235918

**Published:** 2019-11-25

**Authors:** Jose Gustavo De la Ossa, Francesca Felice, Bahareh Azimi, Jasmine Esposito Salsano, Maria Digiacomo, Marco Macchia, Serena Danti, Rossella Di Stefano

**Affiliations:** 1 Cardiovascular Research Laboratory, Department of Surgical, Medical and Molecular Pathology and Critical Care Medicine, University of Pisa, 56126 Pisa, Italy; josegustavo.delao@student.unisi.it (J.G.D.l.O.); francesca.felice77@hotmail.it (F.F.); 2 Doctoral School in Life Sciences, University of Siena, 53100 Siena, Italy; ja.espositosalsano@student.unisi.it; 3 Department of Civil and Industrial Engineering, University of Pisa, 56122 Pisa, Italy; b.azimi@ing.unipi.it; 4 Consorzio Interuniversitario Nazionale per la Scienza e Tecnologia dei Materiali (INSTM), 50121 Florence, Italy; 5 Department of Pharmacy, University of Pisa, 56126 Pisa, Italy; maria.digiacomo@unipi.it (M.D.); marco.macchia@unipi.it (M.M.); 6 Interdepartmental Research Center “Nutraceuticals and Food for Health”, University of Pisa, 56100 Pisa, Italy

**Keywords:** olive leaf extract (OLE), polyphenol, endothelial cells, oxidative stress, in vitro model, scaffold, tissue engineering, biowaste, pruning, poly(vinylidene fluoride tri-fluoroethylene)

## Abstract

Olive leaf extract (OLE) can be obtained as biowaste and is extensively used a food supplement and an over-the-counter drug for its beneficial effects. New studies have investigated OLE concerning the role of oxidative stress in the pathogenesis of vascular disease. This in vitro study aims to evaluate if OLE extracted from the Tuscan *Olea europaea* protects endothelial cells against oxidative stress generated by reactive oxygen species (ROS). Methods: OLE total polyphenols (TPs) were characterized by the Folin–Ciocalteu method. Endothelial cells were grown in conventional cultures (i.e., two-dimensional, 2D) and on a biomaterial scaffold (i.e., three-dimensional, 3D) fabricated via electrospinning. Cell viability and ROS measurement after H_2_O_2_ insults were performed. Results: OLE TP content was 23.29 mg GAE/g, and oleuropein was the principal compound. The dose-dependent viability curve highlighted the absence of significant cytotoxic effects at OLE concentrations below 250 µg/mL TPs. By using OLE preconditioning at 100 µg/mL, cell viability decrease was observed, being in 3D lower than in the 2D model. OLE was protective against ROS in both models. Conclusions: OLE represents a high-value antioxidant source obtained by biowaste that is interesting for biomedical products. Using a 3D scaffold could be the best predictive model to mimic the physiological conditions of vascular tissue reaction.

## 1. Introduction

*Olea europaea* is one of the most ancient trees of the Mediterranean region. Olive leaves, an agricultural waste by-product obtained during the harvesting or pruning process of olive fruit, contain considerable bio-phenols as the other parts of olive tree [1]. Olive leaf extract (OLE) is used in traditional medicine as a food supplement and an over-the-counter drug for a variety of beneficial effects, including anti-inflammatory and anti-atherosclerotic ones [1,2]. 

Recent studies have been conducted on potentials effects of OLE on blood pressure, plasma lipids, inflammatory markers, and on the role of oxidative stress in the pathogenesis of vascular disease [3,4]. The imbalance in the reactive oxygen species (ROS) is known to produce damage to the otherwise healthy tissue [5,6]. Therefore, the use of antioxidant molecules is considered an efficient strategy to counteract the effects of ROS. Antioxidants have shown a protective effect against oxidative stress generated by ROS in several experimental models by scavenging the free radicals, preventing lipid peroxidation, and increasing the expression of antioxidant genes [7,8]. OLE contains active components with potential antioxidant activity. It is suggested that these properties are related to the H-atom donation from the phenolic groups present in the OLE.

Dried OLE is rich in water-soluble phenolic compounds, specifically secoiridoid oleuropein (about 17%), whereas the remaining compounds (apigenine-7-O-glucoside, luteolin-7-O-glucoside, quercetin, and caffeic acid) are contained in substantially smaller amounts (<0.1%) [9]. Numerous studies indicate the therapeutic effect of polyphenol olive derivatives, such as oleuropein and tyrosol, on vascular and endothelial functions by suppressing the intracellular production of ROS [10,11]. 

Novel approaches that can ameliorate antioxidant supplementation based on tissue engineering (TE) are currently of special clinical interest [12,13]. Indeed, by using biocompatible materials in the form of three-dimensional (3D) scaffolds for cell growth and differentiation, it is possible to obtain in vitro biological constructs that resemble, in terms of morphology and function, the native tissues more closely than conventional bi-dimensional (2D) cell cultures [14]. As a consequence, 3D tissue-engineered constructs represent valuable models to assess the effect of new drugs in a more reliable manner [15]. A number of biomaterials and technologies have been used to create TE scaffolds. Among those approved by the Food and Drug Administration for biomedical devices, poly(vinylidene fluoride) (PVDF) and its main copolymers, such as poly(vinylidene fluoride-*co*-trifluoroethylene) (P(VDF-TrFE)), are biostable in chemically aggressive environments (e.g., resistant to most chemicals, solvents, ultraviolet and nuclear radiations, among others), cost-effective, thermally stable, easy processable and lightweight. PVDF can be processed via electrospinning to obtain fibrous nonwoven meshes made up of ultrafine size (few µm down to tens of nm) fibers [16]. The highly porous structure of such meshes and the small diameter of the fibers is invoked to mimic the extracellular matrix (ECM) of biological tissues and thus appears ideal to work as a TE scaffold [16]. In order to be translated into clinical applications, systems based on engineered fiber meshes as an ECM model with the ability to grow cells retaining their functionality, are of great interest for preclinical evaluation [17,18].

This study aims at characterizing OLE as obtained from the discarded leaves of Tuscan olive trees and evaluating its antioxidant effect on in vitro models of endothelial cells, including conventional 2D cultures and innovative 3D scaffolds, to show the potential of biowaste in biomedical applications. In particular, a randomly oriented fiber scaffold was fabricated via electrospinning using P(VDF-TrFE) for the growth of human umbilical vein endothelial cells (HUVECs). This copolymer was chosen for its chemical inertia, which allows in vitro tests with aggressive agents, such as hydrogen peroxide, to be performed without altering the scaffold material. Protection of OLE from oxidative stress induced by hydrogen peroxide is thus assessed. 

Understanding the beneficial effects of OLE will support the use of agrifood waste for high-value products, such as bioactive agents for biomedicine, thus contributing to the generation of sustainable value chains. 

## 2. Results

### 2.1. Characterization of OLE from Tuscan Olea Europaea 

The total polyphenol (TP) content of OLE was determined by the method of Folin–Ciocalteu assay, thus obtaining 23.29 mg gallic acid equivalent (GAE)/g. Moreover, the identification of phenolic compounds of OLE was performed by high performance liquid chromatography (HPLC) analysis. The results showed that oleuropein was the major compound present in the extract with a concentration of 14.69 ± 0.92 mg/g of OLE, corresponding to 1.47 % (*w/w*%). Furthermore, the OLE contained luteolin 7-*O*-glucoside but at lower concentrations of 3.60 ± 0.25 mg/g of OLE, corresponding to 0.36% (*w/w*%). We obtained different quantification of TP in diverse sampling periods (Table 1). TP content obtained by olive leaves harvested between February 2018 and March 2019 ranged between 14.99–27.83 mg/g of OLE extract, the highest content being during springtime.

### 2.2. Scaffold Characterization

**Figure 1:**
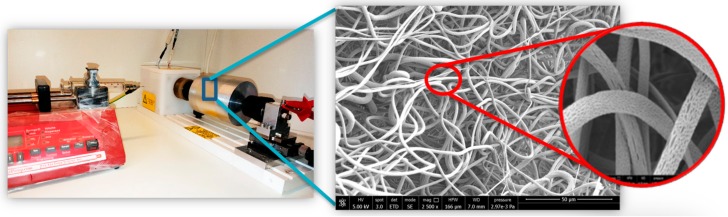
Electrospinning process and fiber morphology. Lens on the right shows nanoporosity on the fiber surface.

Figure 1 shows the electrospinning process and a representative SEM image of the obtained P(VDF-TrFE) scaffolds. Uniformly distributed and bead-less ultrafine fibers were formed under the applied working conditions. Trapped Methyl Ethyl Ketone (MEK) with moisture due to ambient relative humidity (46%) led to the formation of surface nanoporosity (lens in Figure 1), which is desirable in TE, as it offers enhanced surface area for protein deposition and cell adhesion. The 500 rpm rotative collector resulted in randomly oriented fibers with an average diameter of 1.9 ± 0.5 µm and 94% ± 2% porosity. 

### 2.3. Dose- and Time-Dependent Effect of OLE on 2D Culture Model

**Figure 2:**
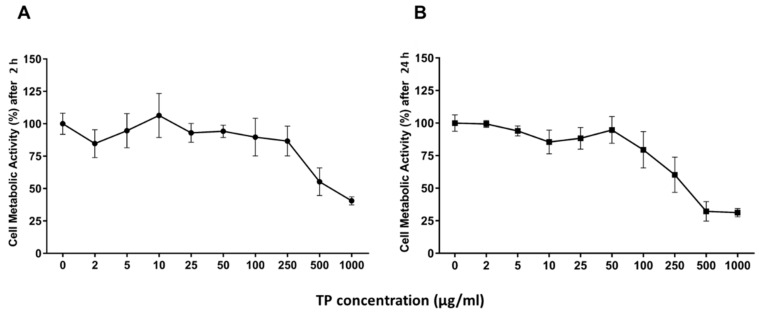
Dose- and time-dependent cell metabolic activity. HUVECs were cultured for 2 h (**A**) and 24 h (**B**) in the presence of different concentrations of TPs from OLE. Cell metabolic activity was determined by WST-1 colorimetric assay and expressed as metabolic activity percentage compared to control (untreated cells). Graphical data are represented as mean ± SD of three separate experiments run in triplicate.

The metabolic cell activity of HUVECs cultured on tissue culture plastics (2D model) was evaluated by WST-1 assay, as described in Section 4.7.1. The dose-dependent metabolic curve showed no significant cytotoxic effects at OLE concentration below 250 µg/mL TP (Figure 2A). To evaluate the time-dependent metabolic activity of HUVECs, a WST-1 assay was performed after 24 h of treatment (Figure 2B). OLE also resulted as non-toxic after a long period of treatment below 250 µg/mL TP. 

### 2.4. Antioxidant Activity of OLE in 2D Culture Model

**Figure 3:**
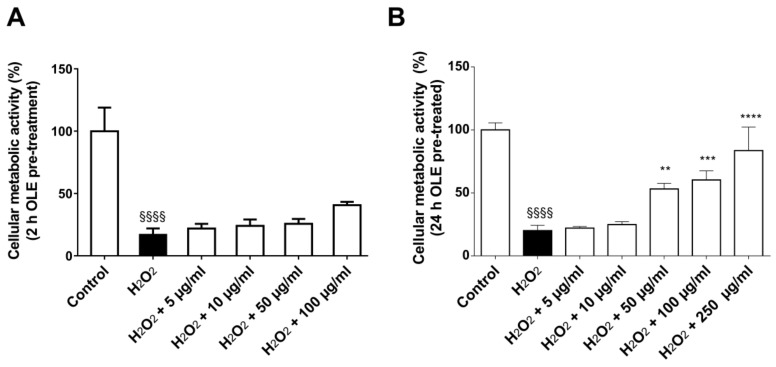
Antioxidant effect of OLE. HUVEC viability was evaluated after 2 h (**A**) and 24 h (**B**) of pre-treatment with different concentrations of OLE (i.e., 0, 5, 10, 50, 100, up to 250 µg/mL of TPs) followed by treatment with 100 µM H_2_O_2_ for 1 h. Data are expressed as metabolic activity percentage compared to control (untreated cells) and are representative of 3 separate experiments run in triplicate. ** *p* < 0.005, *** *p* < 0.0005 and **** *p* < 0.00005 vs. H_2_O_2_; ^§§§§^ *p* < 0.00001 vs. control.

To evaluate the antioxidant activity of OLE, cells were pre-treated for 2 and 24 h with different OLE concentrations, and after that, an oxidative stress insult was applied by treating the cells with 100 µM H_2_O_2_ for 1 h. As shown in Figure 3, only 24 h pre-incubation TP at a concentration range between 50 µg/mL and 250 µg/mL prevented H_2_O_2_-induced viability reduction of HUVECs (*p* < 0.05), thus demonstrating to possess an antioxidant effect. 

### 2.5. ROS Production in 2D Culture Model

**Figure 4:**
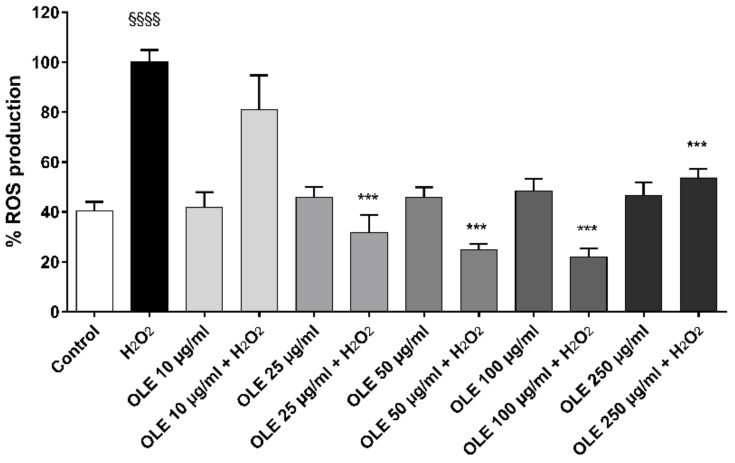
ROS production by HUVECs was evaluated after 24 h of incubation with different concentrations of OLE (i.e., 0, 10, 25, 50, 100, up to 250 µg/mL of TPs) and 100 µM H_2_O_2_ for 1 h. Data are expressed as ROS production% by treated cells and are representative of 3 separate experiments run in triplicate (*** *p* < 0.05 vs. H_2_O_2_; ^§§§§^ *p* < 0.0001 vs. control).

ROS accumulation in HUVECs was evaluated after 24 h pre-treatment of OLE at different concentrations (ranging 10–250 µg/mL of TP). Figure 4 shows the OLE’s effect on ROS accumulation in HUVECs, as determined by CM-H2DCFDA. The results highlighted a significantly protective effect by OLE polyphenols at a concentration below 250 µg/mL TP (*p* < 0.0001 vs. H_2_O_2_).

### 2.6. 3D Culture Model Characterization

**Figure 5:**
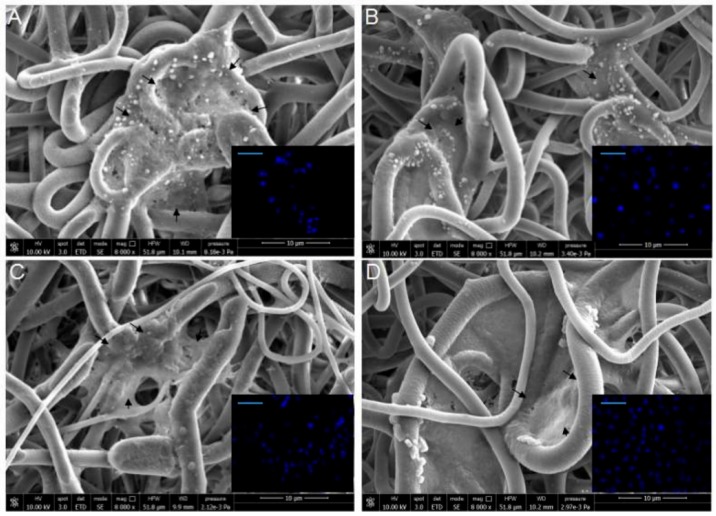
SEM and fluorescence (in inserts) analyses of P(VDF-TrFE)/HUVEC construct: (**A**) cell/scaffold construct (control); (**B**) cell/scaffold construct + OLE; (**C**) cell/scaffold construct + H_2_O_2_; and (**D**) cell/scaffold construct + OLE + H_2_O_2_. Black arrows indicate the HUVECs in the construct. The scale bar in inserts is 100 µm.

SEM was used to evaluate HUVEC morphology and fluorescence microscopy to assess the cellular colonization of the scaffolds by detecting blue-stained cell nuclei (Figure 5). Endothelial cells were grown within the fibrous matrix of the scaffold and showed a good spatial distribution. Cell nuclei were observed at different depths, without any signs of pyknosis, thus corroborating the results obtained with the alamarBlue^®^ test.

### 2.7. OLE Effect on Metabolic Activity in 3D Culture Model 

**Figure 6:**
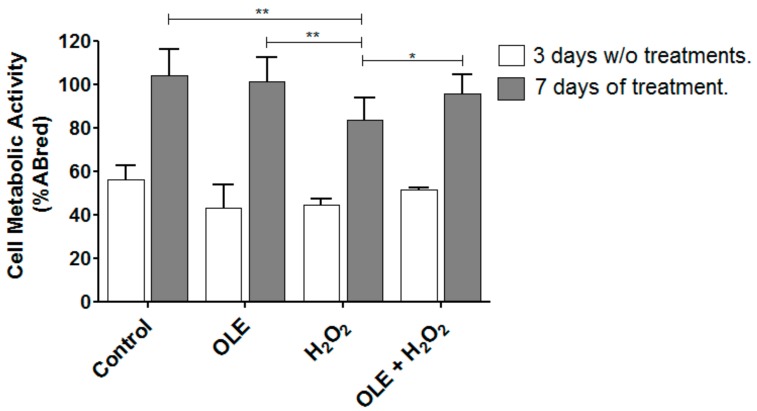
Metabolic activity of HUVECs on P(VDF-TrFE) scaffolds and antioxidant activity of OLE on cell/scaffold constructs. HUVECs after 3 days of culture were viable in the 3D model (white bars) before OLE and H_2_O_2_ treatments. Gray bars represent cell/scaffold constructs incubated with OLE (100 µg/mL of TPs) for 24 h and 100 µM H_2_O_2_ for 1 h. Data are expressed as % alamarBlue^®^ reduction and are representative of 3 separate experiments in triplicate. * *p* < 0.05 and ** *p* < 0.005 vs. H_2_O_2_.

The cytocompatibility of HUVECs grown on P(VDF-TrFE) fiber meshes was analyzed after 3 and 7 culture days using the alamarBlue^®^ assay. As displayed in Figure 6 (white bars), the HUVECs were viable on the scaffolds, showing a metabolic activity above 40%. HUVECs were grown on the scaffolds for 6 days, then they were preconditioned with OLE for 24 h and subsequently administrated with 100 µM H_2_O_2_ for 1 h. The results demonstrated a metabolic activity higher than 80% (grey bars), confirming the protective effect of OLE at a TP dose of 100 µg/mL (* *p* < 0.05 vs. H_2_O_2_).

### 2.8. OLE Effect on ROS in 3D Culture Model 

**Figure 7:**
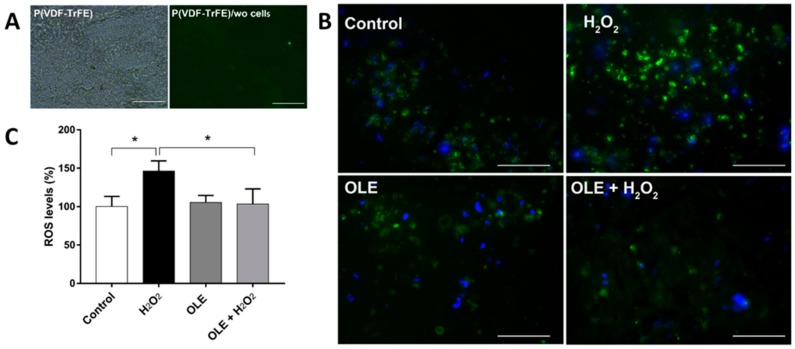
Results of ROS analysis performed on the 3D model treated with OLE for 24 h prior to H_2_O_2_ incubation for 1 h. (**A**) Imaging of fibers without (wo) cells under transmission and fluorescence microscopy modes. (**B**) Panel of fluorescence micrographs of the 3D models under the different treatments showing nuclei in blue and ROS in green. Magnification is 20×; scale bar is 100 µm. (**C**) ROS induction percentage as from integrated OD (* *p* < 0.05 vs. control, H_2_O_2_, and OLE + H_2_O_2_).

ROS production in cell/scaffold constructs was evaluated after 24 h pre-treatment with OLE with 100 µg/mL of TPs, followed by 100 µM H_2_O_2_ incubation for 1 h. Figure 7B,C shows a significantly protective effect of OLE on ROS accumulation in the 3D model of H_2_O_2_-stressed HUVECs (*p* < 0.05). 

The generation of ROS was qualitatively detected using fluorescent microscopy and quantitatively evaluated using ImageJ analysis, as described in the Section 4 Materials and Methods. Briefly, HUVECs were cultured on P(VDF-TrFE) fiber scaffolds for 7 days and then incubated with OLE at 100 μg/mL of TPs for 24 h and 100 µM H_2_O_2_ for 1 h. The live samples were stained with Hoechst 33342 for visualization of cell nuclei (in blue). The generation of ROS (in green) was qualitatively and quantitatively assessed (*n* = 3). 

## 3. Discussion

OLE can be extracted from the waste leaves of the olive tree and represents a valid source of polyphenols. Several studies are currently evaluating the possible applications of OLE, not only for nutrition as a food supplement but also as a pharmaceutical agent, mainly for cardiovascular diseases. Indeed, the antioxidant and cytoprotective effects of OLE have been recognized at the endothelium level. They are of great importance since tissue damage at the vascular wall leads to stress in the vascular endothelium and induces complications in the vascular system by oxidative stress [19]. 

The mechanism by which OLE is capable of exerting such a strong antioxidant activity is a subject of investigation. OLE is rich in polyphenols, mainly in oleuropein, which is its best-known polyphenol. Other polyphenols in OLE have been reported to exert pro-oxidant effects, due to their iron-and-copper-reducing activities. These reduced metals, in turn, can catalyze the production of OH· radical by the Fenton reaction. Moreover, the capacity of dietary polyphenols to act as antioxidant/prooxidants under in vitro and in vivo systems is dependent on a number of factors, such as their concentration and structure [20,21]. These bioactive compounds have shown cell reinforcement, antihypertensive, antiatherogenic, anti-inflammatory, hypoglycemic, and hypocholesterolemic properties [22]. Therefore, there is a growing scientific interest in disclosing, with improved specificity, the potential of OLE in biomedicine. In particular, the Tuscan autochthonous variant of the olive tree (*Olea europaea var. Olivastra seggianese*) is studied for its peculiar properties. Olive oil plantations produce several wastes, mostly unexploited, such as olive tree prunings, which produce, upon burning by farmers, a considerable amount of green house gases [23]. Under a circular economy and life cycle analysis point of view, a sustainable use of agrifood waste to produce high-value products is greatly encouraged in place of arbitrary disposal and lop burning. Solvents commonly used, such as ethanol or aqueous-based ethanol or methanol solutions are generally used for obtaining extracts with high phenol content and antioxidant activity. However, these popular extraction methods suffer from some drawbacks, such as insufficient recovery of extracts and long extraction times, and intensive heating and/or mixing results in high levels of energy consumption [24]. In order to have a fully green process, we opted for water-based extraction. Indeed, the use of water alone has given results comparable to those obtained with ethanol [25]. 

The aim of this study is to characterize OLE from Tuscan olive trees and assess its antioxidant properties. In particular, we set-up a 3D in vitro model by using a biocompatible fibrous scaffold to obtain more reliable results about OLE effects. 3D in vitro tissue models offer ethically sustainable alternatives for drug testing, largely catching the attention of the scientific community. The protective effect of OLE could widen the possibility of the application of agrifood waste derivatives to biomedical devices and TE. 

There are differences in the levels and types of phenolic compounds in *Olea europaea* L. leaves, fruits, and seeds. TP and the chemical composition of olive leaves change according to several conditions, such as origin, the proportion of branches on the tree, storage conditions, climatic conditions, moisture content, and degree of contamination with soil [26]. Our OLE polyphenol characterization showed a TP content of 23.29 mg GAE/g, with oleuropein as a main component. In line with other varieties of *O. europaea*, the TP content in aqueous extraction was around 9–17 mg GAE/g [27]. These results are in accordance with those reported in other studies of polyphenol quantification in OLE [27,28]. However, we obtained different quantification of TPs in diverse sampling periods, with early spring OLE being the richest in TPs.

We investigated the protective effect of OLE from Tuscan olive trees on endothelial cells, using a conventional culture (2D model) and a novel tissue-engineered culture (3D model) of HUVECs. Our past work has, in fact, highlighted the importance of scaffold architecture in generating complex vascularized in vitro models using HUVECs [29]. In this study, a new 3D model was obtained by fabricating a fiber mesh of P(VDF-TrFE), a biocompatible polymer used for biomedical devices and tissue engineering [30]. The fibrous character of the scaffold produced via electrospinning had the purpose of mimicking natural ECM, thus helping the maintenance of native cell morphology and function [17,18]. P(VDF-TrFE) was specifically chosen for scaffold fabrication for its chemical inertia and non-degradability, in order to prevent any biomaterial damage following hydrogen peroxide administration. P(VDF-TrFE) possesses additional peculiar characteristics, such as piezoelectricity, which can be exploited to obtain truly functional 3D models of blood vessels [30,31,32]. It has been outlined that tissue-engineered in vitro models are more reliable than conventional 2D cell cultures on tissue culture plastics [15]. Therefore, we tested the non-cytotoxic dose and ROS protective effect of OLE in 2D and 3D cellular cultures. 

In the 2D model, we demonstrated a dose-dependent effect of OLE on cell metabolic activity: a concentration of 250 µg/mL TPs was sufficient both to reduce the effect of oxidative stress induced by hydrogen peroxide and to produce cytoprotective effects in HUVECs. 

In the 2D and 3D models, we investigated if HUVECs were still protected by OLE after exposure to oxidative stress induced by H_2_O_2_. Following treatment with OLE at 100 µg/mL, we also showed that in the 3D model, OLE exerted cytoprotective effects. The metabolic activity of cells grown on the scaffolds and treated with OLE, followed by H_2_O_2_ treatment, was indeed significantly higher than that observed in OLE-untreated samples. These results demonstrate the indirect antioxidant potential of OLE, possibly acting through the augmentation of cellular antioxidant capacity by enhancing specific genes encoding antioxidant proteins [33]. Moreover, studies have suggested that the combination of olive phenolic compounds, such as those present in OLE, exhibit a synergistic behavior towards free radical elimination, superior to the antioxidant capacity of the vitamin C and E [34]. On the other hand, the drop in metabolic activity experienced by the same cells after the H_2_O_2_ insult in the 2D model was much more enhanced than in the 3D model. This outcome is meaningful since it shows that the 3D model is better predictive of real tissue response, including disease tissue conditions [35]. Ultimately, our results performed on the 3D models confirmed a significantly protective effect of OLE on ROS accumulation in HUVECs, the detected ROS level in OLE pre-treated H_2_O_2_-stressed samples being in the same range of non-stressed control samples, where it was significantly lower than that of H_2_O_2_-stressed samples. Moreover, different from the 2D model in which a 250 µg/mL OLE dose was necessary, in the 3D model, a 100 µg/mL OLE dose was sufficient to have a ROS-protective effect. This can be linked to the reduced metabolic activity drop and may deal with diffusion gradients and other complex phenomena occurring in 3D microenvironments, which are still a subject of investigation [35]. 

The strategy of using natural polysaccharides and proteins to reduce free radical production has been explored in wound healing using plant and milk derivatives such as *Aloe vera* and whey protein [36,37]. Scaffolds loaded with palm extracted polyphenols have shown the ability to reduce free radical-derived inflammation, thus maintaining homeostasis and endothelial cell function [38]. These encouraging results also highlight the increasing scientific interest in the application of characterized natural extracts to biomedicine.

Our results indicate that OLE sustains endothelial cell viability in 3D scaffolds, thus suggesting a potential use in TE applications. For example, many TE scaffolds are obtained by processing aliphatic polyesters, such as polylactic acid, which are highly hydrophobic. On the other hand, polyphenols have demonstrated to enhance cell proliferation on those materials by virtue of their hydrophilic properties. From our results, the polyphenols in the 2D and 3D models might have played an active role in improving cell viability and proliferation. We show that the antioxidant property of polyphenols can both minimize ROS-induced cell damage and improve cell viability. As a next step, biobased scaffolds, incorporating OLE inside the polymer to enable a controlled polyphenol release, could be developed to minimize the oxidative stress and be used in different tissue regeneration fields, such as cardiovascular disease and wound healing.

## 4. Materials and Methods

### 4.1. Materials

HPLC-grade acetonitrile and methanol were purchased from Labscan (Dublin, Ireland). Acetic acidof analytical grade (assay >99.5%) was purchased from Fluka (Buchs, Switzerland). Water was purified by using a Milli-Q System (Millipore, Bedford, MA, USA). Other reagents unmarked were of analytical grade. Methanol (MeOH) was purchased from Carlo Erba (Rodano, MI, Italy), H_2_O_2_ was bought from Farmac-Zabban S.p.a. (Calderara di Reno, BO, Italy), NaCO_3_, gelatin and Hoechst 33342 were obtained from Sigma-Aldrich (Milan, Italy), poly(vinylidenefluoride-*co*-trifluoroethylene) [P(VDF-TrFE)] powder (70:30 % mol) was purchased from Piezotech Arkema (Pierre-Benite, France), Methyl ethyl ketone (MEK) was bought from Merck (Darmstadt, Germany). Medium 199 (M199), fetal bovine serum (FBS), penicillin-streptomycin solution, L-glutamine, HEPES buffer were supplied by Hospira S.r.l. (Naples, Italy). 

4-[3-(4-iodophenyl)-2-(4nitrophenyl)-2H-5-tetrazolium]-1,3-benzenedisulfonate (WST-1 assay), was purchased from Roche Applied Science (Mannheim, Germany), 5-(and-6)-chloromethyl-2’,7’-dichloro-di-hydro-fluorescein diacetate, acetyl ester (CM-H_2_DCFDA) was supplied by Invitrogen. EGM-2 medium bullet kit was bought from Lonza (Verviers, Belgium), alamarBlue^®^ was obtained from Thermo Fisher Scientific (Waltham, MA, USA).

### 4.2. Sample Preparation

Olive leaves were obtained from *Olivastra seggianese* groves. The collection was performed at CNR-IVALSA, Follonica (GR), Italy. Leave collection was carried out manually and at different months between February 2018 and March 2019. The leaves were stored at 25 °C after harvesting. For each lot, 20 g of leaves were placed in liquid nitrogen and crushed manually. A final weight ratio of 0.9 g OLE/g (namely, 18 g of OLE powder per 20 g of olive leaves) was obtained and used for future experiments. 

### 4.3. OLE Polyphenol Characterization

To measure TP content, 1 mg of OLE powder was dissolved in water and therefore analyzed with Folin–Ciocalteau’s method [39], as per the manufacturer’s instructions. The results were expressed in gallic acid equivalent (GAE), a universally accepted standard for polyphenols to determine the value of the TP in 100 g of sample. 

### 4.4. HPLC Characterization 

A high-performance liquid chromatography analysis (HPLC) was carried out to identify and quantify the major phenolic compounds of the OLE using a slightly modified method developed in our previous study [40].

The retention times and UV absorbance spectra of phenolic compounds present in OLE were compared with those of the commercial standard and quantified at 278 nm, using p-hydroxyphenyl acetic acid as the internal standard, according to the previously reported method. Sample concentrations were determined by linear regression. For each calibration curve, the correlation coefficients were >0.99. 

HPLC analysis was performed using an HPLC instrument (Beckman, Ramsey, MN) equipped with a System Gold Solvent Delivery module (Pumps) 125 and a System Gold UV/Vis Detector 166, set to 280 nm, and using a Phenomenex Gemini reverse-phase C18 column (250 × 4.6 mm, 5 μm particle size; Phenomenex, Castel Maggiore, Italy). The mobile phase was a mixture of H_2_O/AcOH (97.5:2.5 *v/v*) (A) and MeOH/ACN (1:1 *v*/*v*) (B), programmed as follows: a gradient from 5% (B) to 30% (B) in 45 min; 30% (B) for 5 min and then from 30% (B) to 5% (B) in 5 min. The flow rate was 1 mL/min, and the injected volume was 50.0 μL.

### 4.5. Endothelial Cell Isolation and Culture 

Tissue samples were collected and treated anonymously and in conformity with the Declaration of Helsinki. Human umbilical vein endothelial cells (HUVECs) were isolated from the umbilical vein endothelium of healthy human donor cords, following the procedure described by Jaffe et al. [41]. Isolated HUVECs were centrifuged, the cell pellet was plated on gelatin pre-coated flasks and incubated for 24 h at 37 °C, 5% CO_2_ using growth culture medium consisting of M199 (PAN-Biotech), supplemented with 10% FBS, penicillin-streptomycin solution, l-glutamine, HEPES buffer. After 24 h, the growth medium was replaced to remove excess red blood cells.

### 4.6. Scaffold Fabrication and Characterization 

P(VDF-TrFE) was dissolved in MEK at a concentration of 20% *w/v* and stirred at 300 rpm for 12 h at room temperature to allow complete dissolution. The polymer solution was loaded into a 10 mL glass syringe, fitted with a blunt tip stainless steel needle (21G × 3/4”), and placed into a syringe pump (NE-300, New Era Pump Systems, Inc., NY, USA). The ground terminal of high voltage supply (S1600079 Linari High Voltage, Linari Engineering s.r.l., Pisa, Italy) was connected to the metal needle, whereas the positive terminal was connected to the collector; 35 kV potential was applied. A cylindrical collector (diameter 8 cm, Linari Engineering s.r.l.), was placed at a distance of 15 cm from the tip of the needle. The polymer solution was injected from the needle in the presence of the electric field at a constant flow rate of 0.016 mL/min and collector velocity of 500 rpm, room temperature, and 46% relative humidity. The fiber meshes were kept in an oven at 60 °C overnight to remove solvent traces. The morphology of the scaffolds was evaluated by scanning electron microscopy (SEM) using an FEI FEG-Quanta 450 instrument (Field Electron and Ion Company, Hillsboro, OR, USA). The samples were sputtered with gold (Gold Edwards SP150B, UK) before analysis. 

### 4.7. Investigation of OLE Effects

#### 4.7.1. 2D HUVEC Model

HUVECs between passage P2–P4 were treated for 2 and 24 h with different polyphenol concentrations of OLE (2 μg/mL, 5 μg/mL, 10 μg/mL, 25 μg/mL, 50 μg/mL, 100 μg/mL, 250 μg/mL, 500 μg/mL, 1000 μg/mL) in growth medium with 5% FBS (*n* = 3).

Cell viability was determined by colorimetric assay with WST-1, based on the cleavage of tetrazolium salt by mitochondrial dehydrogenases, present in viable cells. Briefly, at the end of the treatment, HUVECs were incubated with tetrazolium salt at 10 μL/well for 3 h at 37 °C, in 5% CO_2_ cell culture incubator. Thereafter, the formazan dye formed was quantified by measuring the absorbance at 450 nm, with a multiplate reader (Thermo Scientific Multiskan FC microplate photometer). The absorbance was directly correlated to the number of metabolically active cells, and viability was expressed as a percentage of viable cells. 

The protective effect of OLE was evaluated pre-treating HUVECs with different OLE polyphenol concentrations for 2 and 24 h and then with 100 μM of H_2_O_2_ for 1 h. At the end of each treatment, cells were analyzed for viability and ROS production.

Intracellular ROS production was evaluated by ROS fluorescent probe CM-H_2_DCFDA, a cell-permeable indicator for these compounds. Briefly, during the last 30 min of treatment with H_2_O_2_, the HUVECs were co-incubated with CM-H_2_DCFDA at 10 μM/well dissolved in PBS, in the dark at room temperature. ROS production was detected by measuring the increase in fluorescence over time by microplate reader (Thermo Scientific Fluoroskan Ascent Microplate Fluorometer), using excitation at 488 nm and emission at 510 nm.

#### 4.7.2. 3D HUVEC Model 

The P(VDF-TrFE) scaffolds were cut into 0.8 × 0.8 mm squares and sterilized in absolute ethanol overnight. The scaffolds were coated with a sterile-filtered 2% (*w/v*) gelatin aqueous solution for 30 min and seeded with HUVECs at a density of 1 × 10^5^ cells/sample. Cell/scaffold constructs were cultured in EGM-2 medium bullet kit (CC-4176, Lonza, Walkersville, MD, USA) for 1 week in a standard incubator (37 °C, 5% CO2) by replacing the medium every 48 h. 

The in vitro effect of OLE on metabolic activity and ROS production was tested on HUVEC/scaffold constructs following H_2_O_2_ administration, according to the following experimental design (*n* = 3):

- Cell/scaffold construct (control);
- Cell/scaffold construct + OLE (100 µg/mL GAE) for 24 h;
- Cell/scaffold construct + H_2_O_2_ (100 µM) for 1 h;
- Cell/scaffold construct + OLE (100 µg/mL GAE for 24 h) + H_2_O_2_ (100 µM for 1 h).

Cell metabolic activity was assessed using the alamarBlue^®^ assay, according to the manufacturer’s instructions and expressed as the percentage of reduced alamarBlue^®^ (%ABred). Data were acquired after 3 days of culture without treatments to evaluate cytocompatibility and post-treatment to evaluate the antioxidant activity of OLE. The absorbance of supernatants was measured with a spectrophotometer (Victor 3; PerkinElmer, Waltham, MA, USA) under a double-wavelength reading (570 and 600 nm). Finally, %ABred was calculated by correlating the absorbance values and the molar extinction coefficients of the dye at the selected wavelengths, following the protocol provided by the manufacturer. 

At the endpoint, the cell/scaffold constructs were washed twice with 1× PBS and then fixed in 1% (*w/v*) neutral buffered formalin for 10 min at 4 °C. SEM was used on cell/scaffold constructs to image cell attachment, for morphology, and for spreading onto the scaffolds. The biological samples were dehydrated with 70% ethanol for 30 min and finally dried in oven at 37 °C for two h prior to sputter coating. For examination, the samples were mounted on aluminum stubs, sputter-coated with gold, and observed under SEM. 

Intracellular ROS production was evaluated by ROS fluorescent probe CM-H2DCFDA. Briefly, after cell treatment, the cells/scaffold constructs were washed twice with 1× PBS and then stained with Hoechst 33342 (5 µM for 10 min) for nuclei visualization. The constructs were then washed twice with 1 × PBS and incubated with 10 μM CM-H2DCFDA in PBS for 30 min in the dark at room temperature. The cells/scaffold constructs were then washed three times with PBS and visualized under a fluorescent microscope (Carl Zeiss MicroImaging GmbH, Germany). ImageJ software (Version 1.52r) was used to measure the integrated optical density (OD) of the stained cells, which was normalized for green + blue integrated OD (*n* = 3).

### 4.8. DAPI Staining

Formalin-fixed samples were washed with 1× PBS and incubated with 10 g/mL 4’,6-diamidino-2-phenylindole (DAPI-Life Technologies, acquired by Thermo Fisher Scientific, Waltham, MA, USA) in 1x PBS for 10 min RT, to detect cell nuclei (fluorescent in blue) and washed in 1× PBS. Specimens were observed by an inverted fluorescence microscope equipped with a digital camera (Nikon Eclipse Ti, Amsterdam, The Netherlands).

### 4.9. Statistical Analysis

Data are presented as mean ± standard deviation (SD). Three independent replications were evaluated for each treatment with OLE. The in vitro experiments were performed in three separate series of three independent replicates for each well. The difference among groups of values was evaluated by a one-way ANOVA, and a post hoc analysis was performed by Tukey’s or Bonferroni’s multiple comparisons test when appropriated. Differences with a *p*-value < 0.05 were considered statistically significant.

## 5. Conclusions

OLE was obtained from the leaves of the Tuscan *Olea europaea* after prunings, namely 18 g OLE/20 g leaves, and characterized. It resulted highly rich in polyphenols (23.29 mg GAE/g), among which oleuropein accounted for a concentration of 14.69 ± 0.92 mg/g of OLE corresponding to 1.47% (*w*/*w*%), and luteolin 7-*O*-glucoside for a lower concentration of 3.60 ± 0.25 mg/g of OLE, corresponding to 0.36% (*w*/*w*%).

OLE administration to endothelial cells (HUVECs) showed a protective effect against ROS production, which was also confirmed in a 3D culture model. This model was set up under the tissue engineering approach by using electrospun P(VDF-TrFE) scaffolds. OLE showed good cytocompatibility and antioxidant activity, which revealed effectiveness in controlling the oxidative stress on HUVECs upon exposure to an H_2_O_2_ insult_._ A number of medical applications can potentially benefit from OLE, including cardiovascular disease and wound healing, especially for wounds compromised by ROS stress. Local administration of OLE may thus represent a valid and strategy in biomedicine, which is also compliant with a sustainable use of bio-resources.

## Figures and Tables

**Table 1:** Quantification of TP in olive leaf lyophilized extract in different periods.

Sampling Period (Month-Year)	TP (mg/g)
02–2018	27.83
05–2018	23.06
10–2018	14.99
03–2019	23.29

## Abbreviations

AcOH	Acetic Acid	
ANOVA	Analysis of variance
CM-H2DCFDA	5-(and-6)-chloromethyl-2’,7’-dichloro-di-hydro-fluorescein diacetate, acetyl
DAPI	4’,6-Diamidino-2-phenylindole dihydrochloride
ECM	Extracellular matrix
EGM-2	Endothelial Cell Growth Medium
FBS	Fetal Bovine Serum
GAE	Gallic Acid Equivalent
H_2_O_2_	Hydrogen Peroxide
HEPES	(4-(2-hydroxyethyl)-1-piperazineethanesulfonic acid)
HPLC	High Performance Liquid Chromatography
HUVEC	Human Umbilical Vein Endothelial Cells
M199	Medium 199
MeOH	Methanol
MEK	Methyl Ethyl Ketone
Na_2_CO_3_	Sodium Carbonate
OLE	Olive Leaf Extract
PVDF	Poly(vinylidene fluoride)
ROS	Reactive Oxygen Species
SD	Standard deviation
SEM	Scanning Electronic Microscopy
P(VDF-TrFE)	Poly(vinylidenefluoride-*co*-trifluoroethylene)
TE	Tissue Engineering
TP	Total Polyphenol
UV	Ultraviolet
WST-1	4-[3-(4-iodophenyl)-2-(4nitrophenyl)-2H-5-tetrazolium]-1,3-benzenedisulfonate ester
